# Advances in reproductive management strategies for thyroid cancer patients during the perinatal period: a narrative review

**DOI:** 10.3389/fonc.2025.1642792

**Published:** 2025-09-01

**Authors:** Ying Dong, Juxiang Gou

**Affiliations:** Department of Thyroid Surgery, West China Hospital, Sichuan University, Chengdu, Sichuan, China

**Keywords:** thyroid cancer, perinatal period, fertility, management strategies, narrative review

## Abstract

Changes in thyroid function during the perinatal period significantly impact patients’ fertility, necessitating targeted adjustments in management strategies to ensure hormone stability while weighing the effects of treatment on both the mother and the fetus. Therefore, comprehensive management during the perinatal period is essential to promote effective reproductive outcomes. This study aims to review the relationship between thyroid cancer and fertility, focusing on reproductive management strategies during the preconception, pregnancy, and postpartum periods in patients with thyroid cancer. Additionally, we will synthesize this information into illustrative figures to provide clinical healthcare professionals with a theoretical foundation for improving reproductive care for thyroid cancer patients throughout the perinatal period, thereby facilitating comprehensive reproductive health management.

## 1 Introduction

**Figure 1:**
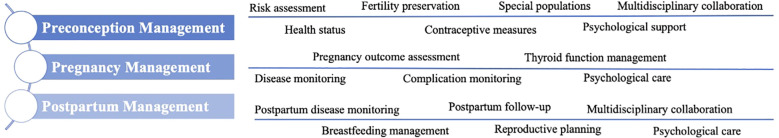
Diagram of reproductive management strategies for thyroid cancer during the perinatal period.

Thyroid cancer accounts for 20% of all diagnosed cancers during the perinatal period, ranking as the second most common cancer after breast cancer (1). As one of the most prevalent endocrine malignancies among women of reproductive age, thyroid cancer presents complex clinical challenges. Physiological changes during pregnancy may impact disease diagnosis and treatment, while hormonal fluctuations (such as elevated estrogen and human chorionic gonadotropin) potentially accelerate tumor growth and increase management complexity. Therapeutic interventions like Radioactive Iodine Therapy (RAIT) may adversely affect ovarian reserve and fertility (2).The diagnosis and subsequent treatment of thyroid cancer negatively influence the reproductive intentions of nearly 40% of women, with 40% deciding not to have children and 33% experiencing anxiety about medicalized pregnancy (3). Compounded by patients’ reluctance to undergo radiological or invasive procedures during pregnancy and the typically mild or non-acute clinical symptoms, approximately two-thirds of thyroid cancer diagnoses occur within the first 12 months postpartum (4).The psychological and social pressures associated with pregnancy planning and potential parenthood are intensified, consequently reducing the quality of life for young women (5, 6). The decision-making regarding disease treatment and fertility, the balancing of multiple roles (patient, expectant mother, family roles), as well as the healthcare needs during pregnancy all emphasize the importance of comprehensive reproductive health management and protection for patients during the perinatal period (7, 8). However, there has been no in-depth analysis on this topic in the current literature. Therefore, this study aims to review the reproductive management strategies for thyroid cancer during the perinatal period from the aspects of preconception, pregnancy, and postpartum (see **Figure 1**). This review will provide clinical healthcare professionals with a theoretical foundation for better reproductive management of thyroid cancer patients, thereby promoting comprehensive reproductive health protection.

## 2 Relationship between thyroid cancer and fertility

### 2.1 Impact of pregnancy on thyroid cancer

Pregnancy itself does not significantly increase the risk of thyroid cancer progression; however, the timing of pregnancy and the patient’s disease status critically influence prognosis. A large-scale study by Van Velsen et al. (9) involving 1,505 patients revealed no significant differences in overall disease progression rates between pregnant (12.1%) and non-pregnant groups (14.4%). Structural progression rates (9.3% vs. 10.4%) and biochemical progression rates (2.8% vs. 4.0%) also showed no statistically significant variations. Xiao et al. (10) further confirmed these findings through a study of 311 reproductive-age patients, demonstrating no significant differences in disease progression, tumor enlargement, or lymph node metastasis between pregnant and non-pregnant groups. A systematic review and meta-analysis by Shan et al. (11) found a recurrence rate of 13% among pregnant patients, with no significant difference compared to non-pregnant groups. Even patients with distant metastases exhibited similar outcomes. However, the authors emphasized that the timing of pregnancy and the patient’s disease state are crucial. Patients becoming pregnant less than one year after treatment showed a significantly increased risk of disease progression. Colombo et al. (12) conducted a long-term follow-up study with an average of 97 months, revealing that even for patients with persistent biochemical or structural disease, the disease state could remain relatively stable during pregnancy. Notably, patients with distant metastases (such as pulmonary metastases) did not show apparent disease deterioration during pregnancy. An important consideration is the physiological changes in thyroid function during pregnancy, which complicate disease monitoring. In iodine-sufficient regions, thyroid gland volume increases by approximately 10%, while in iodine-deficient areas, this increase can reach 20-40%. Serum thyroglobulin concentrations rise during pregnancy, particularly in iodine-deficient conditions, potentially affecting follow-up assessments for patients with residual lesions after hemithyroidectomy or initial treatment.

### 2.2 Impact of thyroid cancer and its treatment on fertility

Thyroid cancer and its treatment may affect patients’ fertility, primarily manifesting as increased infertility rates and prolonged time to first pregnancy, with limited impact on pregnancy outcomes. However, surgical-related complications, especially hypoparathyroidism, may bring special management challenges. A large-scale study by Hirsch et al. (13) involving 1,164 thyroid cancer patients and 5,030 control subjects found that patients’ infertility rate (23.9%) was significantly higher than the control group (20.4%), with the median time from diagnosis to first pregnancy extended by 6 months (37 months vs. 31 months). Although patients receiving RAIT had similar overall pregnancy rates to those without treatment (46.9% vs. 47.7%), patients receiving multiple RAIT treatments experienced a significantly prolonged time to first pregnancy. A study by Cao et al. (14) on 96 thyroid cancer survivors showed no significant differences in adverse pregnancy outcomes such as premature birth, pregnancy-induced hypertension, gestational diabetes, and miscarriage compared to 18,236 control subjects. Thyroidectomy and RAIT did not significantly increase the risk of adverse pregnancy outcomes, and even the time interval between treatment and pregnancy had no significant impact on pregnancy outcomes. However, surgical-related complications may bring special management challenges, with approximately 10% of surgical patients potentially experiencing permanent hypoparathyroidism, requiring calcium supplementation and/or active vitamin D. Even with stable calcium concentrations, changes in calcium and vitamin D metabolism during pregnancy may lead to hypocalcemia or hypercalcemia during pregnancy and lactation (15). Insufficient or excessive treatment of hypoparathyroidism during pregnancy may lead to serious complications such as miscarriage, stillbirth, and perinatal death. Maternal hypocalcemia can cause fetal parathyroid hyperplasia and skeletal changes, while maternal hypercalcemia may suppress fetal parathyroid hormone production, causing neonatal hypocalcemia (16).

## 3 Management strategies for thyroid cancer during the periconceptional period

### 3.1 Preconception management

#### 3.1.1 Fertility-related risk assessment

Reproductive history in thyroid cancer patients is closely associated with disease risks, including miscarriage history, fertility history, high-risk reproduction, menstrual history, and hormonal factors. Nursing personnel need to strengthen high-risk population screening through systematic assessment to provide a basis for personalized management. A large-scale study by Jin et al. (17) involving 1,303 thyroid cancer patients and 106,602 control subjects found that hysterectomy and bilateral oophorectomy were significantly associated with thyroid cancer risk in young women, while increased fertility might have a protective effect. This finding was further validated by Abe et al. (18) in a multi-ethnic cohort study (n=118,344), which indicated that oophorectomy and hysterectomy were risk-increasing factors, while first live birth age ≤20 years was a protective factor. Notably, Zervoudis (19) studied thyroid and breast cancer comorbidity and discovered that miscarriage history and multiple pregnancies were important risk factors. In the dual cancer group, 81% of patients had a miscarriage history, significantly higher than 62% in the pure breast cancer group. Nursing personnel must pay special attention to patients’ pregnancy outcome history during assessment. He (20) investigated the association between reproductive and menstrual factors and Papillary Thyroid Cancer (PTC) in Chinese women. The study found that early menarche, short-term breastfeeding, and pre-menopausal status might be associated with PTC occurrence, while earlier first pregnancy age could potentially reduce PTC risk. Understanding the impact of reproductive and menstrual factors on PTC risk helps identify high-risk populations and optimize screening strategies. For high-risk patients not completely in remission before pregnancy, nurses should assist doctors in developing a more rigorous monitoring plan, including regular checks of serum thyroglobulin levels and neck ultrasound (9).

#### 3.1.2 Disease status assessment and counseling

Preconception assessment needs to focus on disease status and biomarker levels to predict disease progression risk, thereby developing personalized management plans. Van Velsen (9) through a study of 1,505 thyroid cancer patients found that patients reaching complete remission before pregnancy had a lower disease progression risk (9.6%), while patients with structural incomplete remission had significantly higher risk (33%), and the disease status needs to be fully considered when planning pregnancy. Colombo (12) confirmed that even for DTC patients with persistent biochemical or structural disease, pregnancy is usually safe, with low disease progression risk. For DTC patients planning pregnancy, especially those with persistent disease status, a detailed clinical assessment should be conducted, including disease staging, risk stratification, and dynamic risk assessment. Patients should be informed that there may be a slight risk of biochemical or structural disease progression during pregnancy, but the overall prognosis is good. Explaining the potential impacts of pregnancy on the disease to patients helps alleviate their anxiety and enables informed decision-making.

#### 3.1.3 Treatment timing and fertility preservation strategies before treatment

The selection of treatment timing and the protection of fertility require careful consideration. The impact of Radioactive Iodine Therapy (RAIT) on fertility deserves special attention, which necessitates appropriate protective measures and reasonable time arrangements. Van Velsen (2) conducted an in-depth study on the importance of treatment timing selection, proposing that although hormonal changes during pregnancy might influence thyroid cancer growth, their impact on disease progression is limited. The study recommends waiting at least 6–12 months after RAIT before planning pregnancy. Piek (21) provided a biological basis for this finding, discovering that RAIT dose-dependently reduces serum anti-Müllerian hormone levels, directly affecting fertility. For patients requiring RAIT, fertility preservation measures such as egg or embryo freezing should be considered.

#### 3.1.4 Contraceptive measures and fertility preservation after treatment

Reasonable contraceptive measures are an important component of preconception management for thyroid cancer patients, as pregnancy during RAIT may pose serious teratogenic risks to the fetus. A cross-sectional study by Milla (22) provided detailed data on contraceptive status, showing that 75.4% of patients receiving RAIT used contraception, with intrauterine devices (28.6%) being the most common method. 71.7% of patients primarily obtained contraceptive information from doctors, emphasizing the importance of contraceptive counseling. Special attention should be given to education and guidance for patients not using contraceptive measures to ensure pregnancy prevention during treatment. Hirsch (13) suggested that for patients undergoing RAI treatment, special attention should be paid to changes in ovarian function and fertility, with strengthened follow-up and assessment of delayed first pregnancy, and provision of corresponding support. For young thyroid cancer patients, healthcare personnel should advocate for fertility preservation measures before treatment, such as egg or embryo freezing, to safeguard patients’ reproductive choices.

#### 3.1.5 Multidisciplinary collaborative management and lifestyle guidance

Management of thyroid cancer during the periconceptional period requires multidisciplinary collaboration and emphasis on lifestyle interventions, with nursing personnel playing a crucial role in coordinating various specialties and guiding patients’ lifestyle improvements. The clinical practice guidelines by Anonymous (7) emphasize establishing a multidisciplinary collaborative system involving endocrinology, obstetrics and gynecology, and oncology departments, providing specific recommendations across screening, diagnosis, surgery, and post-operative management. The guidelines particularly highlight the importance of patient education and communication, with nurses assisting patients in understanding the assessment process for thyroid nodules during screening and diagnostic stages. Furthermore, Klobodu (23) explored the role of lifestyle interventions for cancer patients facing fertility challenges. The study found that patients encounter multiple barriers when attempting to improve dietary behaviors, including lack of nutrition-related resources, work-life balance challenges, and treatment-related fatigue. However, the research also discovered that healthcare providers’ support and patients’ motivation for change are important facilitating factors. Patients are more likely to trust health recommendations from doctors or nutritionists and have a strong awareness of additional health benefits of a healthy diet. Therefore, in preconception management, in addition to routine medical interventions, patient lifestyle guidance and health education should be given significant attention.

#### 3.1.6 Psychological support and health education

Thyroid cancer patients during the periconceptional period often face dual pressures of disease treatment and fertility needs, requiring appropriate psychological support and health education. Li (24) conducted a quantitative study on 218 post-thyroid cancer surgery women of reproductive age, systematically assessing their fertility concerns. The study found the total fertility concern score was 55.65 ± 7.31, with self-health and children’s health being the primary concern dimensions. Fertility intention, number of children, education level, post-surgery time, and social support were the main factors influencing concern levels. Van Velsen (2) and Klobodu (23) further emphasized the importance of psychological support, indicating that an effective social support system can significantly alleviate patients’ fertility concerns. They suggested conducting fertility assessment early after surgery and providing focused psychological support for patients without children or with fertility intentions, while helping patients establish and maintain an effective social support network. Ge J (25) conducted in-depth interviews with 12 young female thyroid cancer patients with fertility plans, identifying four themes: concerns about fertility, treatment risks to the fetus, worry about disease progression, and lack of fertility safety information. They emphasized that reproductive issues should not be overlooked when studying and focusing on thyroid cancer diagnosis and treatment, as they significantly impact patients’ quality of life. Healthcare workers need to strengthen communication and discussion about fertility issues with young female thyroid cancer patients. Van Velsen (9) further proposed that nurses should provide scientific evidence to DTC patients about pregnancy’s disease progression risk, helping patients alleviate anxiety about potential recurrence through clear communication and reassurance regarding similar risks of adverse pregnancy outcomes (6, 26, 27).

#### 3.1.7 Preconception management for special populations

Infertile patients are a high-risk group requiring special attention, facing not only a higher risk of thyroid cancer but also needing more comprehensive nutritional management and psychological support. Ding (28) analyzed data from the Taiwan National Health Insurance Research Database and found that infertile women had a significantly higher thyroid cancer incidence rate compared to the control group (2.85/10,000 person-years vs. 1.53/10,000 person-years), with risk notably increasing in patients followed up for over 7 years. The use of fertility medications might further elevate this risk. Nursing personnel should conduct detailed health risk assessments for infertile women and provide education about thyroid cancer risks, helping them understand the potential long-term health impacts of infertility and fertility medications. Tan L (29) also pointed out that whether thyroid tumors affect female fertility is related to their impact on thyroid function. For infertile patients with thyroid tumors, after determining the tumor’s benign or malignant nature, assisted reproduction should be considered based on specific circumstances, followed by tumor treatment.

### 3.2 Pregnancy management

#### 3.2.1 Disease monitoring and progression assessment

Disease monitoring for thyroid cancer patients during pregnancy requires a systematic and personalized approach, focusing on treatment timing, disease status, and dynamic biochemical indicator changes, establishing a comprehensive monitoring system. Van Velsen (9) found through a large-sample study that while overall disease progression risk showed no significant difference (12.1% vs 14.4%), the risk significantly increased for patients becoming pregnant within one year after treatment. Colombo et al. (12) conducted a long-term follow-up study (average 97 months) and further discovered that even patients with persistent biochemical or structural disease could maintain disease stability through close monitoring, a finding applicable to patients with distant metastases. Nursing personnel should focus on regularly assessing biochemical indicators during monitoring, including thyroglobulin and anti-thyroglobulin antibody levels. More frequent follow-up should be conducted for patients with structural incomplete remission (progression risk 33%), establishing personalized monitoring plans to ensure timely detection of disease changes. Pregnancy has minimal impact on recurrence risk for previously treated differentiated thyroid cancer (DTC) patients. For patients with good treatment response before pregnancy (complete or uncertain response), pregnancy need not be delayed, and no special monitoring is required during pregnancy. For patients with incomplete treatment response (biochemical or structural incomplete response), enhanced disease monitoring during pregnancy is necessary (11).

#### 3.2.2 Pregnancy outcome assessment and risk prevention

Thyroid cancer patients generally have good pregnancy outcomes, but specific treatments and disease states may increase the risk of certain complications, requiring targeted prevention and monitoring. Kim (30)found that patients who received Radioactive Iodine Therapy (RAIT) compared to those who underwent only surgery had a higher risk of adverse pregnancy outcomes, with a notably increased risk of premature birth for patients becoming pregnant within 6 months after RAIT. Spiegel et al. (31), through a large-scale study of 14,513,587 pregnant women, discovered that thyroid cancer patients were more likely to require blood transfusions and experience venous thromboembolism. However, the impact on neonatal outcomes such as congenital malformations, intrauterine growth restriction, and premature birth was limited. Nursing personnel need to focus on preventing and monitoring pregnancy complications, particularly assessing the risk of thromboembolism.

#### 3.2.3 Complication monitoring and prevention

While thyroid cancer patients generally have good pregnancy outcomes, specific treatments and disease states may increase the risk of certain complications, requiring targeted prevention and monitoring. Orloff (15) elaborated on prevention and treatment strategies for hypocalcemia, emphasizing that nursing personnel should monitor patients’ calcium and parathyroid hormone levels, and assist in implementing preventive measures, including optimizing vitamin D levels and protecting parathyroid blood supply. Lebrun (16) further noted that hypocalcemia and hypoparathyroidism during pregnancy, though rare (75% originating from surgery), can lead to serious complications such as premature birth, miscarriage, and stillbirth. Therefore, clinical medical personnel need to specifically adjust treatment plans throughout pregnancy to meet calcium requirements for the fetus, newborn, and mother. Key focuses should include: Regular monitoring of blood calcium and parathyroid hormone levels, Adjusting calcium supplementation plans according to different pregnancy stages, Observing maternal and infant status, Timely detecting early signs of complications. Li’s research (32) found that pregnancy has minimal impact on serum thyroglobulin levels and lymph node metastasis in differentiated thyroid cancer (DTC) patients, suggesting that physiological thyroid function changes during pregnancy have limited interference with disease monitoring.

#### 3.2.4 Thyroid function management during pregnancy

Thyroid function changes significantly during pregnancy, requiring targeted management strategies to ensure hormone level stability while balancing treatment plans’ impact on mother and child. Spiegel (31) evaluated the association between thyroid cancer and maternal-fetal outcomes during pregnancy, aiming to understand the impact of thyroid cancer diagnosis before or during pregnancy on pregnancy complications and neonatal outcomes, proposing that thyroid cancer patients can achieve similar outcomes to women without the disease under appropriate management. Colombo (12) observed patients with persistent biochemical or structural disease and found that thyroid function indicators might fluctuate during pregnancy but can remain relatively stable under appropriate management. All patients in the study maintained normal reproductive function during the average follow-up period, with smooth pregnancies and deliveries, and no fetal complications. Anonymous (7) emphasized through clinical practice guidelines that TSH suppression treatment during pregnancy requires dynamic adjustment, with target values varying at different stages. Excessive suppression or functional decline may affect pregnancy outcomes. Colombo (12) further discovered that even in patients with pulmonary metastasis, disease status remained stable during pregnancy through appropriate monitoring and management, highlighting the importance of multidisciplinary collaboration.

#### 3.2.5 Psychological care during pregnancy

Thyroid cancer patients during pregnancy face unique psychological pressures, requiring systematic assessment and timely intervention to help patients establish positive disease coping strategies. The diagnosis and treatment of thyroid cancer during pregnancy present special challenges, necessitating a balance between maternal-fetal safety and disease treatment. Patients often experience psychological burden due to the initially asymptomatic nature of the disease. Research (33) indicates that patients commonly express concerns about potential disease progression due to surgical delays and are troubled by treatment timing choices, worrying about the disease’s impact on their family, especially those who incidentally discover thyroid abnormalities during prenatal examinations. Rooney (27) confirmed through a prospective cohort study (n=501) that persistent psychological stress may reduce pregnancy success rates by up to 29%. Endocrine system disease patients particularly face dual pressures from both the disease itself and fertility-related stress. Nursing personnel should conduct systematic psychological assessments, provide personalized stress management skill training, implement cognitive behavioral therapy and relaxation techniques, and refer to professional psychological counseling when necessary.

### 3.3 Postpartum management

#### 3.3.1 Postpartum disease monitoring and assessment

The postpartum period is a critical phase for managing thyroid cancer patients, requiring attention to disease progression risks, thyroid function changes, and hormone level regulation, and establishing a systematic monitoring system. Shan et al. (11) reported through meta-analysis that the overall postpartum disease recurrence rate was 13%, especially emphasizing the necessity of developing stratified management strategies based on patient treatment response. Regarding monitoring indicators, Zhang et al. (34) revealed specific changes in thyroid function during the postpartum period through a longitudinal study, finding that early postpartum periods may experience temporary functional fluctuations, with more pronounced changes among breastfeeding patients. Bath et al. (35) discovered through systematic tracking that thyroglobulin levels, as an important monitoring indicator, vary with breastfeeding status and iodine intake, recommending dynamic and personalized monitoring approaches during the postpartum period. Kitahara et al. (36) pointed out that hormonal changes during pregnancy might increase maternal thyroid cancer risk by affecting thyroid cell proliferation and differentiation. Attention should be paid to high-risk pregnancies (such as large fetal birth weight, postpartum hemorrhage, severe pregnancy vomiting), and early screening of thyroid function and thyroid diseases should be conducted for these patients.

#### 3.3.2 Breastfeeding management strategies

Breastfeeding management requires special attention to the impact of Radioactive Iodine Therapy (RAIT), providing scientific breastfeeding guidance while ensuring patient treatment needs. Alexander et al. (37) provided detailed guidelines for postpartum thyroid disease management, specifically emphasizing RAIT’s impact on breastfeeding, recommending cessation of breastfeeding 6 weeks to 3 months before RAIT. Lee et al. (33) noted that thyroid cancer patients face unique challenges during the postpartum period, needing to balance breastfeeding intentions, treatment timing, and parental responsibilities. They suggested that nursing personnel provide more professional and personalized guidance to help patients make reasonable decisions. Due to pregnancy and breastfeeding affecting calcium and vitamin D metabolism, up to 10% of patients may experience permanent hypoparathyroidism after thyroid surgery (15, 38). Even with normal and stable calcium concentrations, hypocalcemia or hypercalcemia may occur, requiring calcium and/or active vitamin D replacement therapy (16). Moreover, maternal hypocalcemia can lead to fetal parathyroid hyperplasia and related skeletal changes, while maternal hypercalcemia may suppress fetal parathyroid hormone production, causing neonatal hypocalcemia. Therefore, serum calcium levels should be closely monitored during pregnancy (e.g., every 3–4 weeks) and breastfeeding period (e.g., monthly), with maintaining normal calcemia as the primary objective (16).

#### 3.3.3 Postpartum follow-up and long-term management

Postpartum follow-up requires a systematic and personalized monitoring plan, adjusting follow-up strategies based on risk stratification and disease status to ensure long-term disease management. Anonymous et al. (7) provided a framework-level guidance in clinical practice guidelines, emphasizing the development of follow-up plans based on disease risk stratification. Low-risk patients may reduce monitoring frequency, while patients with distant metastasis or high-risk diseases should have more frequent follow-ups. Shan (11) discovered through meta-analysis that management needs differ for patients with various treatment response states. For patients with good treatment responses, routine monitoring frequency can be maintained, while those with incomplete treatment responses require intensified postpartum follow-up. The study also detailed postpartum treatment decisions, including whether additional Radioactive Iodine Therapy (RAIT) is necessary and its timing. Furthermore, medical personnel should continue to monitor patients’ biochemical and imaging indicators postpartum to assess disease status changes. For patients requiring long-term TSH suppression therapy, nursing staff should assist in medication management to ensure treatment adherence (12). Continued monitoring of thyroid function, assessment of disease recurrence risk, and particular attention to reproductive health and long-term thyroid function changes for patients who received RAIT are crucial (14).

#### 3.3.4 Reproductive planning guidance

Planning for subsequent pregnancy requires comprehensive consideration of disease status, treatment history, and patient wishes, providing personalized fertility guidance. Liu Qiongfen (39) conducted a cross-sectional survey exploring factors influencing postpartum fertility decisions, finding that disease status and treatment methods are key influencing factors, while also considering individual factors such as patient age, education level, and family support. Wang Rui (40) provided specific recommendations for timing of subsequent pregnancy. Their research discovered that Radioactive Iodine Therapy (RAIT) affects menstrual rhythm in the early stage (within 6 months), but generally returns to normal after 6 months. Based on this finding, they suggested considering pregnancy one year after treatment. Li (32) recommended that patients planning subsequent pregnancy should wait at least one year after treatment to reduce disease progression risk. For patients who have undergone RAIT, Kim (30) advised special attention to the time interval between treatment and subsequent pregnancy. They noted that RAIT is associated with increased risk of adverse pregnancy outcomes, particularly when becoming pregnant within 6 months of treatment. They recommended delaying pregnancy after RAIT to reduce the risk of premature birth and other complications.

#### 3.3.5 Multidisciplinary collaborative management

Postpartum management requires establishing a systematic multidisciplinary collaboration mechanism, providing comprehensive healthcare services through team cooperation. Effective multidisciplinary collaboration is crucial for postpartum management. Anonymous (7) emphasized in their guidelines that postpartum management necessitates joint participation from multiple specialties, including endocrinology, obstetrics and gynecology, and oncology. Nursing personnel play a coordinator role within the team, responsible for information transmission between departments, supervising treatment plan implementation, ensuring follow-up plan execution, and providing continuous health education and psychological support. For patients requiring long-term TSH suppression therapy, the multidisciplinary team should pay special attention to medication adherence, treatment response monitoring, complication prevention, and reproductive function protection. Through systematic team collaboration, the team aims to ensure the continuity and effectiveness of postpartum management.

#### 3.3.6 Postpartum psychological support and health education

Postpartum patients have diverse psychological needs that require systematic assessment and intervention measures, while also addressing the dual pressures of disease management and child-rearing. Bresner et al. (6) found that approximately 42% of patients require support groups, and 43% desire psychological counseling services. Gao WJ et al. (41) conducted a prospective longitudinal study involving 168 patients, revealing that fertility anxiety remained at a moderately high level before discharge and at 1, 3, and 6 months post-discharge, with initial levels of fertility anxiety significantly negatively impacting fertility intentions. Lee et al. (33) highlighted through qualitative research that thyroid cancer patients may be uniquely affected by the disease’s initially asymptomatic nature, potentially influencing their medical-seeking behaviors and postpartum management strategies. Rooney et al. (27) recommended incorporating stress management and cognitive behavioral therapy as standard psychological support measures within treatment protocols. Li (24) further found that women of childbearing age after thyroid cancer surgery generally have high levels of fertility concerns, especially among those who wish to conceive or are childless. The level of social support has a significant alleviating effect on fertility worries. Therefore, it is important to strengthen the social support system for patients in postoperative care, emphasizing the importance of early fertility assessment and psychological support, and it is recommended to incorporate reproductive health into the postoperative care plan.

## 4 Conclusion

This study aimed to review fertility management strategies for thyroid cancer patients during the perinatal period. The findings reveal that preconception management focuses on assessing disease status and reproductive function, requiring rational contraception and fertility plans. Pregnancy management needs special attention to disease monitoring and complication prevention, with regular assessments of thyroid function and biochemical indicators to timely identify and address potential problems. Postpartum management should emphasize disease recurrence monitoring, medication guidance during breastfeeding, and developing long-term follow-up plans.

### 4.1 Implication for future

In the treatment of thyroid cancer, the choice of surgical intervention directly impacts the patient’s fertility. Common surgical types include total thyroidectomy, partial resection, and subtotal resection, which are selected based on the characteristics of the tumor: total resection is appropriate for larger or multifocal tumors, while partial resection is suitable for early localized lesions. Although these surgeries are crucial in treatment, they come with certain risks, such as thyroid dysfunction, vocal cord damage, bleeding, and infection. These complications may affect postoperative recovery and even disrupt the patient’s menstrual cycle and ovulation, further interfering with fertility.

With the development of minimally invasive and robot-assisted surgeries, these new techniques are attracting attention due to their smaller incisions, reduced blood loss, and faster recovery times. Existing studies show that these advanced surgical methods can effectively reduce postoperative complications and improve pregnancy outcomes. Therefore, understanding the indications for surgery, the potential risks, and the advantages of emerging technologies is vital for optimizing fertility management in patients.

The role of nursing staff is equally indispensable, as they not only provide professional preoperative consultations but also conduct comprehensive physical and psychological assessments for patients postoperatively to ensure smooth recovery and implementation of fertility plans. Considering these factors collectively, clinical doctors and nursing teams should collaborate to develop personalized treatment and care plans. When managing the fertility of patients with thyroid cancer, a holistic approach is needed, particularly focusing on key time points such as pre-pregnancy, during pregnancy, and postpartum care. Pre-pregnancy, it is essential to enhance the accuracy of risk assessment tools for pregnancy in patients who have undergone thyroid surgery, aiding doctors in evaluating potential risks, and developing personalized fertility preservation plans for those wishing to retain their fertility. During pregnancy, a systematic pregnancy outcome assessment mechanism should be established, reinforcing interdisciplinary collaboration to ensure that patients receive comprehensive care while providing psychological support to alleviate anxiety. Postpartum, effective monitoring should be implemented, focusing on mental health and thyroid function, offering specific management advice for patients wishing to breastfeed, and guiding their fertility plans.
